# Trait-based approach revealed the seasonal variation of mesozooplankton functional groups in the South Yellow Sea

**DOI:** 10.1007/s42995-022-00156-9

**Published:** 2023-02-13

**Authors:** Zhishuang Zhang, Hongju Chen, Yixuan Li, Ruping Ge, Guangxing Liu, Shaukat Ali, Yunyun Zhuang

**Affiliations:** 1 Key Laboratory of Marine Environment and Ecology, Ministry of Education, Ocean University of China, Qingdao, 266100 China; 2 Marine Ecology and Environmental Science Laboratory, Pilot National Laboratory for Marine Science and Technology, Qingdao, 266200 China; 3 Department of Environmental Sciences, Karakoram International University, Gilgit, 15100 Pakistan

**Keywords:** Zooplankton, Southern Yellow Sea, Functional trait, Functional group, Seasonal variation

## Abstract

Functional traits determine the fitness of organisms and mirror their ecological functions. Although trait-based approaches provide ecological insights, it is underexploited for marine zooplankton, particularly with respect to seasonal variation. Here, based on four major functional traits, including body length, feeding type, trophic group, and reproduction mode, we quantified the seasonal variations of mesozooplankton functional groups in the South Yellow Sea (SYS) in the spring, summer, and autumn of 2018. Strong seasonal dynamics were identified for all traits but patterns varied among traits. Small zooplankton (47.7–88.6%), omnivores–herbivores (81.3–97.6%), and free spawners (54.8–92.5%) dominated in three seasons, while ambush feeders and current feeders dominated in spring (45.7%), and autumn (73.4%), respectively. Cluster analysis of the functional traits showed that the mesozooplankton in the SYS can be classified into eight functional groups. The biogeographic and seasonal variations of functional groups can be partially explained by environmental drivers. Group 1, represented by omnivores–herbivores, was the most dominant functional group, the abundance of which peaked in spring and was positively correlated with chlorophyll *a* concentration, indicating its close association with phytoplankton dynamics. The contribution of giant, active ambush carnivores, passive ambush carnivore jellyfish, current omnivores–detritivores, and parthenogenetic cladocerans increased with sea surface temperature. The proportion of giant, active ambush carnivores and active ambush omnivore–carnivore copepods decreased with salinity in autumn. This study presents a new perspective for understanding the dynamics of zooplankton and paves the way for further research on the functional diversity of zooplankton in the SYS.

**Supplementary Information:**

The online version contains supplementary material available at 10.1007/s42995-022-00156-9.

## Introduction

Zooplankton play a pivotal role in the marine food web by transferring the photosynthetically fixed carbon from primary producers to high-trophic organisms, thereby influencing biogeochemical cycles and energy flow in the marine ecosystem (Buitenhuis et al. 2006; Kiørboe 1997). They are also key players in the biological pump driving carbon from the surface layer to the deep ocean through the production of sinking fecal particles, molting (crustacean exoskeletons), carcasses, and vertical migration (Steinberg and Landry 2017). As bio-indicators (Hays et al. 2005; Taylor et al. 2002), their functional traits, such as size, feeding strategy and reproductive mode, are sensitive to environmental changes, including temperature, salinity, food availability, seasonality, etc. (Kiørboe et al. 2015; Pomerleau et al. 2015; Violle et al. 2007).

Functional traits refer to phenotypic characteristics of organisms that influence their fitness and consequently affect their ecosystem functions (Violle et al. 2007). Species with similar traits could be clustered into a functional group, which refers to a group of species that play similar roles in ecosystem processes (Gitay and Noble 1997). Functional group analysis simultaneously considers multiple traits that could provide a comprehensive insight into the diversity of zooplankton ecological strategies (Litchman et al. 2013), and the response mechanism of zooplankton to environmental changes (Krztoń and Kosiba 2020).

A trait-based approach is well established in marine primary producers (e.g., Edwards et al. 2013) but has not been fully exploited in zooplankton, a major group of marine secondary producers. For example, based on functional traits, Benedetti et al. (2016, 2018) categorized Mediterranean copepods into groups with distinct ecological roles and investigated their distributions along an environmental gradient. Veríssimo et al. (2017) used a trait-based approach to assess the functional diversity of copepod communities in two Brazilian tropical estuaries that have been affected by different degrees of human activity. The biomass anomalies of zooplankton functional groups on the west coast of Vancouver Island, Canada were linked to environmental drivers (Venello et al. 2021). Functional trait-based approaches provide insights into their ecological functions and response to climate change (Benedetti et al. 2019), and have important implications for biodiversity conservation efforts, particularly advances in protected area design (Miatta et al. 2021; Rosenfeld 2002). To the best of our knowledge, a trait-based approach has rarely been used to characterize a zooplankton community in Chinese marginal seas (Li et al. 2022; Zhang et al. 2021), which impedes a better understanding of pelagic ecosystem dynamics and functions.

The South Yellow Sea (SYS) is an important marginal sea of the Western Pacific, and its environment shows seasonal variation and complex hydrologic characteristics, including the Yellow Sea Cold Water Mass (YSCWM, Su and Weng 1994), the Yellow Sea Coastal Current (YSCC), and the Changjiang River Diluted Water (CDW, Wu et al. 2014), all of which significantly influence the dynamics of the zooplankton community (Shi et al. 2015; Sun et al. 2010; Wu et al. 2014). Zooplankton are an important food source for fish; thus, the seasonal dynamics of the zooplankton community are crucial for fishery replenishment in the SYS, which harbors many fishing grounds (Zhang and Jin 2010). The seasonal variations in the community structure and functional groups of zooplankton in the SYS have been widely studied (Shi et al. 2015; Sun et al. 2010; Sun and Sun 2014; Wang et al. 2002). However, functional group identification in previous studies in SYS only considered body size and taxonomic characters (e.g., giant crustaceans, large copepods). Recently, based on four traits, Li et al. (2022) studied the seasonal variations in functional diversity and groups in crustacean zooplankton in the SYS, in which functional groups were hierarchically clustered using a species × trait matrix (Lavorel et al. 1997). This study inspired us to use similar robust methods to explore whether seasonality in terms of functional traits and groups generally exist in mesozooplankton in SYS.

In this study, we tested the hypothesis that seasonality shapes the functional dynamics of zooplankton by characterizing the functional traits and groups of mesozooplankton in the SYS during spring, summer, and autumn of 2018 and elucidating their relationship with physico-chemical variables. Whether a specific ecological strategy would be selected when taxonomic diversity varied among seasons has been discussed. This study presents a new perspective for understanding the dynamics of zooplankton and paves the way for further research on the functional diversity of zooplankton in the SYS.

## Results

### Environmental parameters

**Fig. 1:**
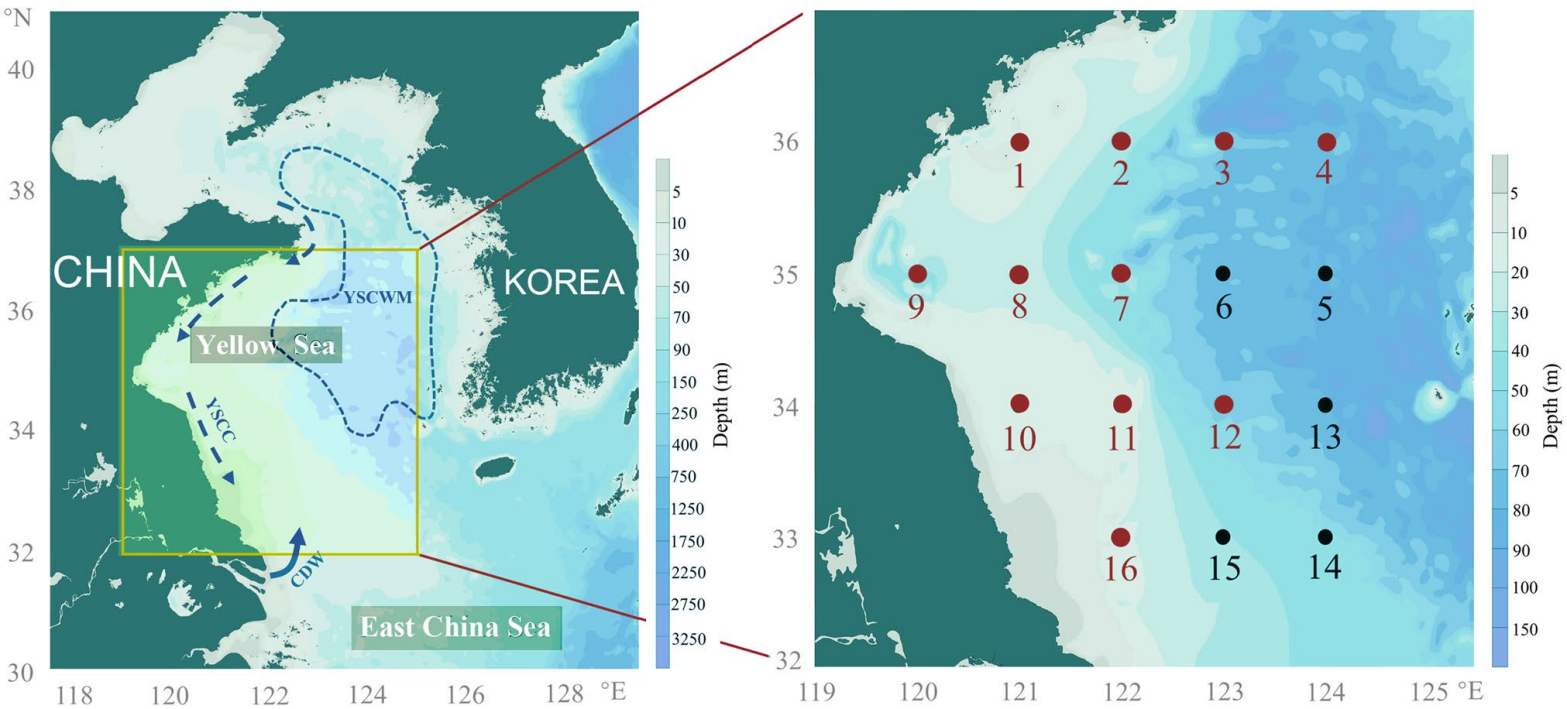
Maps of the study area and sampling sites in the SYS (black sampling sites are not included in spring). In the left figure, the dotted blue arrows indicate the Yellow Sea Coastal Current (YSCC); the solid blue arrow indicates the Changjiang River Diluted Water (CDW); the blue dotted line marks the boundary of the cold center of the Yellow Sea Cold Water Mass (YSCWM) in summer. The circulation patterns were drawn based on previous studies (Su and Weng 1994; Wei et al. 2011; Wu et al. 2014)

**Fig. 2:**
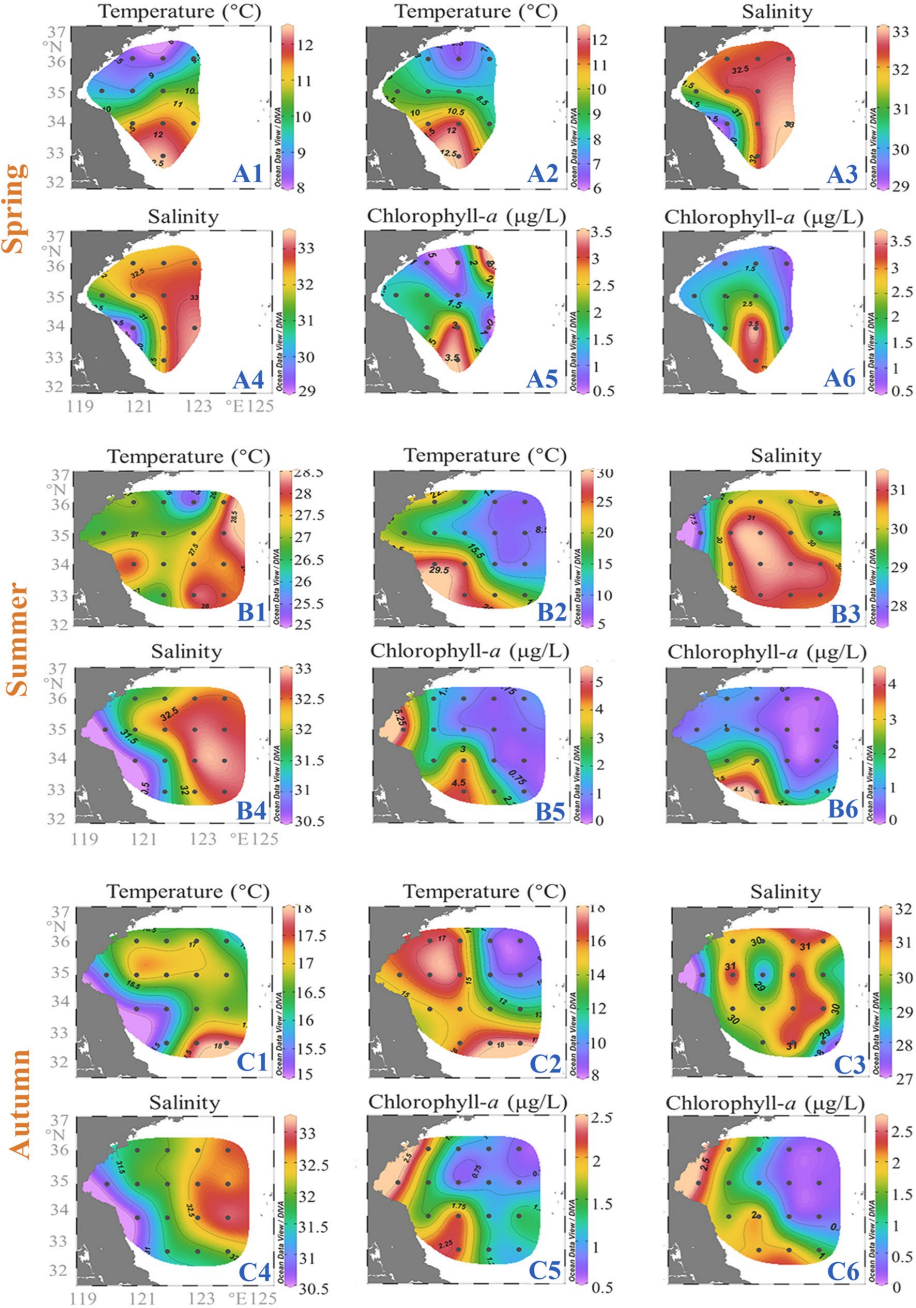
Variations of environmental factors in the South Yellow Sea in three seasons. **A** Spring; **B** Summer; **C** Autumn; 1: sea surface temperature (SST/°C); 2: sea bottom temperature (SBT/°C); 3: sea surface salinity (SSS); 4: sea bottom salinity (SBS); 5: sea surface Chl *a* (S-Chla/μg.L^−1^); 6: sea bottom Chl *a* (B-Chla/μg.L^−1^)

Sampling was carried out at 11, 16, and 16 stations in April, August, and November of 2018 respectively (Fig. 1). The average sea water temperature in the study area varied among the three seasons. Specifically, both the average sea surface temperature (SST) and the average sea bottom temperature (SBT, measured at the depth about 3 m above the bottom) peaked in summer (SST 27.2 ± 0.8 °C; SBT 15.7 ± 7.1 °C), followed by autumn (SST 16.6 ± 0.8 °C; SBT 14.2 ± 3.2 °C) and spring (SST 10.1 ± 1.4 °C; SBT 9.2 ± 1.8 °C) (Fig. 2). SST and SBT gradually decreased from south to north in spring. Owing to the influence of YSCWM, cold water centers (< 10 °C) were present in the bottom layer of the northeastern part of the study area (Fig. 2). The average sea surface salinity (SSS) was higher than the average sea bottom salinity (SBS) in all three seasons (Fig. 2). SSS in spring and SBS in all three seasons showed a trend of low inshore and high offshore salinity (Fig. 2). The average sea surface chlorophyll *a* (S-Chla) was highest in summer (1.84 ± 1.59 μg/L), followed by spring (1.80 ± 1.13 μg/L) and autumn (1.35 ± 0.5 μg/L), while the average sea bottom chlorophyll *a* (B-Chla) peaked in spring (1.87 ± 0.91 μg/L), followed by autumn (1.12 ± 0.83 μg/L) and summer (1.10 ± 1.11 μg/L) (Fig. 2). B-Chla content gradually decreased from south to north in the study area in spring. S-Chla and B-Chla levels were both high in the inshore and low in the offshore (Fig. 2).

### Species composition and abundance

A total of 126 zooplankton taxa (including 25 planktonic larvae) were identified in the SYS during the three seasons with copepod being the most diverse group across seasons (Table 1). The total zooplankton abundance in the SYS generally showed a non-uniform distribution in the study area (Supplementary Fig. S1) and varied among seasons with highest abundances in summer (2102.4 ± 1995.0 ind/m^3^), followed by spring (1568.2 ± 1797.1 ind/m^3^) and autumn (1328.6 ± 1031.5 ind/m^3^). Copepods, chaetognaths, and cladocerans comprised over 80.9% of the total abundance of zooplankton. The average abundance of copepods contributed more than 50% of the total abundance in the three seasons and accounted for more than 80% of the total abundance in autumn. Although the number of hydromedusae species was high in summer and autumn, its contribution to total zooplankton abundance was relatively low (Table 1). The relative abundance of zooplankton showed seasonal and biogeographic variations (Supplementary Fig. S2).

**Table 1:** Composition and abundance of zooplankton species recorded in the South Yellow Sea in three seasons

Taxonomical component	Spring		Summer		Autumn		*P*
	Number of species	Abundance (ind/m^3^)	Number of species	Abundance (ind/m^3^)	Number of species	Abundance (ind/m^3^)	
Hydromedusae	4	1.6 ± 3.5	22	22.3 ± 35.6	13	0.7 ± 1.2	< 0.001
Siphonophorae	**–**	**–**	2	32.4 ± 50.8	3	1.0 ± 2.5	< 0.001
Ctenophores	**–**	**–**	2	0.7 ± 1.7	1	0.3 ± 0.6	0.025
Polychaetes	**–**	**–**	1	< 0.1	**–**	**–**	**–**
Ostracods	**–**	**–**	1	< 0.1	**–**	**–**	**–**
Cladocerans	1	0.3 ± 0.9	2	78.1 ± 271.3	1	0.5 ± 1.2	0.001
Copepods	18	1262.7 ± 1327.5	40	1609.1 ± 1769.2	25	1258.0 ± 990.3	0.752
Amphipods	2	2.2 ± 3.9	3	10.0 ± 18.6	2	6.7 ± 9.9	0.343
Isopods	**–**	**–**	1	< 0.1	1	< 0.1	0.240
Cumaceans	**–**	**–**	1	< 0.1	2	0.1 ± 0.3	0.209
Mysids	**–**	**–**	**–**	**–**	1	0.4 ± 0.9	**–**
Euphausiids	2	0.5 ± 0.8	2	1.8 ± 2.0	2	1.1 ± 2.1	0.499
Decapods	1	< 0.1	3	4.7 ± 8.5	1	0.1 ± 0.2	< 0.001
Chaetognaths	2	6.4 ± 8.0	3	90.0 ± 70.3	3	46.0 ± 55.3	< 0.001
Tunicates	2	8.7 ± 26.7	4	46.9 ± 44.3	3	45.4 ± 64.9	0.009
Pelagic larvae	13	285.9 ± 675.3	22	205.1 ± 545.6	15	50.3 ± 112.9	0.459
Total	45	1568.2 ± 1797.1	109	2102.4 ± 1995.0	73	1328.6 ± 1031.5	0.499

–indicates that the group was not detected

Dominant species were identified according to dominance indicator (*Y*). Twelve dominant species were identified, including eight copepod species, one tunicate species, and three groups of pelagic larvae (Table 2). The copepod *Oithona similis* had the highest dominance in spring with an average abundance of 546.4 ind/m^3^ (Table 2), while *Paracalanus. parvus* had the highest dominance in summer and autumn with an average abundance of 546.9 and 874.9 ind/m^3^, respectively (Table 2).

**Table 2:** Dominance and average abundance of dominant zooplankton species in the South Yellow Sea in three seasons

Dominant species	Spring		Summer		Autumn		*P*
	Abundance (ind/m^3^)	Dominance	Abundance (ind/m^3^)	Dominance	Abundance (ind/m^3^)	Dominance	
*Calanus sinicus*	**202.4 ± 269.3**	**0.11**	**52.8 ± 46.5**	**0.03**	**33.7 ± 36.3**	**0.03**	0.038
*Paracalanus parvus*	**242.1 ± 310.4**	**0.08**	**546.9 ± 316.4**	**0.29**	**874.9 ± 747.4**	**0.64**	0.010
*Centropages dorsispinatus*	**–**	**–**	**320.1 ± 1225.6**	**0.05**	< 0.1	< 0.01	0.003
*Centropages abdominalis*	**208.1 ± 584.1**	**0.09**	**–**	**–**	**–**	**–**	**–**
*Acartia pacifica*	< 0.1	< 0.01	**128.5 ± 366.5**	**0.04**	2.4 ± 6.7	< 0.01	< 0.001
*Oithona similis*	**546.4 ± 989.5**	**0.52**	**198.8 ± 292.8**	**0.12**	**202.8 ± 262.3**	**0.17**	0.363
*Oithona plumifera*	11.2 ± 24.2	< 0.01	**81.4 ± 102.4**	**0.04**	3.8 ± 6.9	< 0.01	0.024
*Corycaeus affinis*	19.6 ± 28.9	0.01	**50.1 ± 41.9**	**0.02**	**86.6 ± 173.2**	**0.02**	0.039
*Oikopleura dioica*	8.5 ± 26.3	< 0.01	**40.8 ± 42.8**	**0.02**	**45.0 ± 64.7**	**0.03**	0.013
Bivalve larvae	**208.0 ± 650.1**	**0.07**	9.5 ± 18.5	< 0.01	16.2 ± 51.2	< 0.01	0.416
Polychaeta larvae	1.1 ± 2.8	< 0.01	**153.2 ± 519.3**	**0.07**	6.3 ± 12.3	< 0.01	0.001
Copepod nauplii	**55.3 ± 57.3**	**0.04**	13.6 ± 25.5	< 0.01	4.9 ± 8.1	< 0.01	0.003

Note: “bold” indicates that the species was dominant (*Y* ≥ 0.02), “**–**” indicates that the species was not detected

### Functional traits and groups of zooplankton

**Fig. 3:**
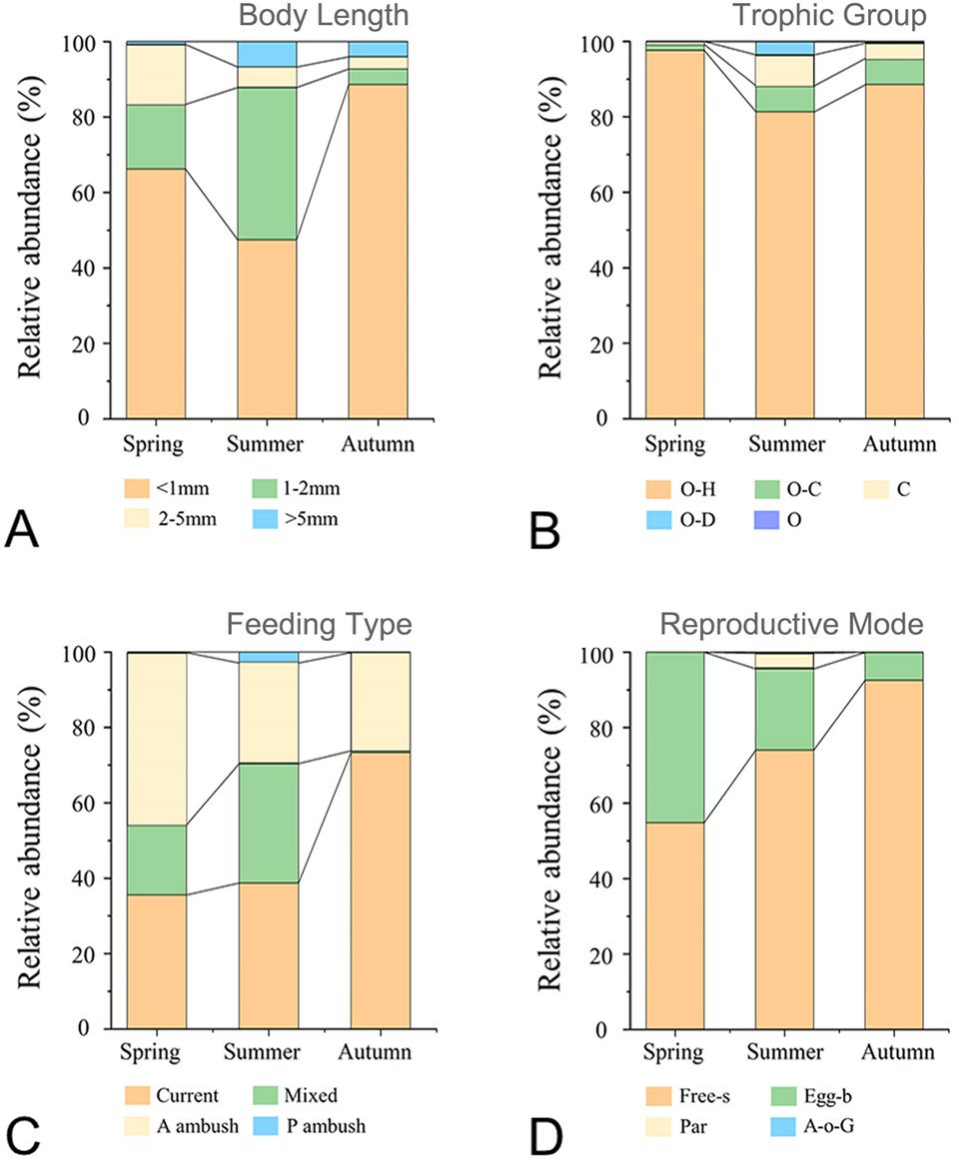
Seasonal variations of four major functional traits. **A** body length; **B** trophic group; **C** feeding type; **D** reproduction mode. O–H: omnivores–herbivores; O–C: omnivores–carnivores; C: carnivores; O–D: omnivores–detritivores; O: omnivores. A ambush: active ambush; P ambush: passive ambush. Free–s: Free spawner; Egg–b: Egg-brooding; Par: parthenogenesis; A–o–G: Alternation of Generations

Four major functional traits, which are relatively stable with the respect to space and time, describing the life cycle and features of zooplankton were analyzed for each taxon, (Benedetti et al. 2016; Kiørboe et al. 2015; Pomerleau et al. 2015). These included body length, trophic group, feeding type and reproductive mode (see “Materials and methods” for details). In this study, significant seasonality was observed in trait modality distribution but the pattern varied among traits (Fig. 3; Supplementary Table S1). Small zooplankton (< 1 mm), omnivores–herbivores and free spawners dominated (> 45%) in three seasons in terms of body length, trophic group and reproductive mode, respectively. Contrastingly, giant zooplankton (> 5 mm), omnivores, passive ambush, parthenogenetic and alternation-of-generations types contributed least (< 7%). Interestingly, for reproductive mode, the relative abundance of free spawners increased with time from spring to autumn, while that of egg brooders showed the opposite trend (Fig. 3D).

**Fig. 4:**
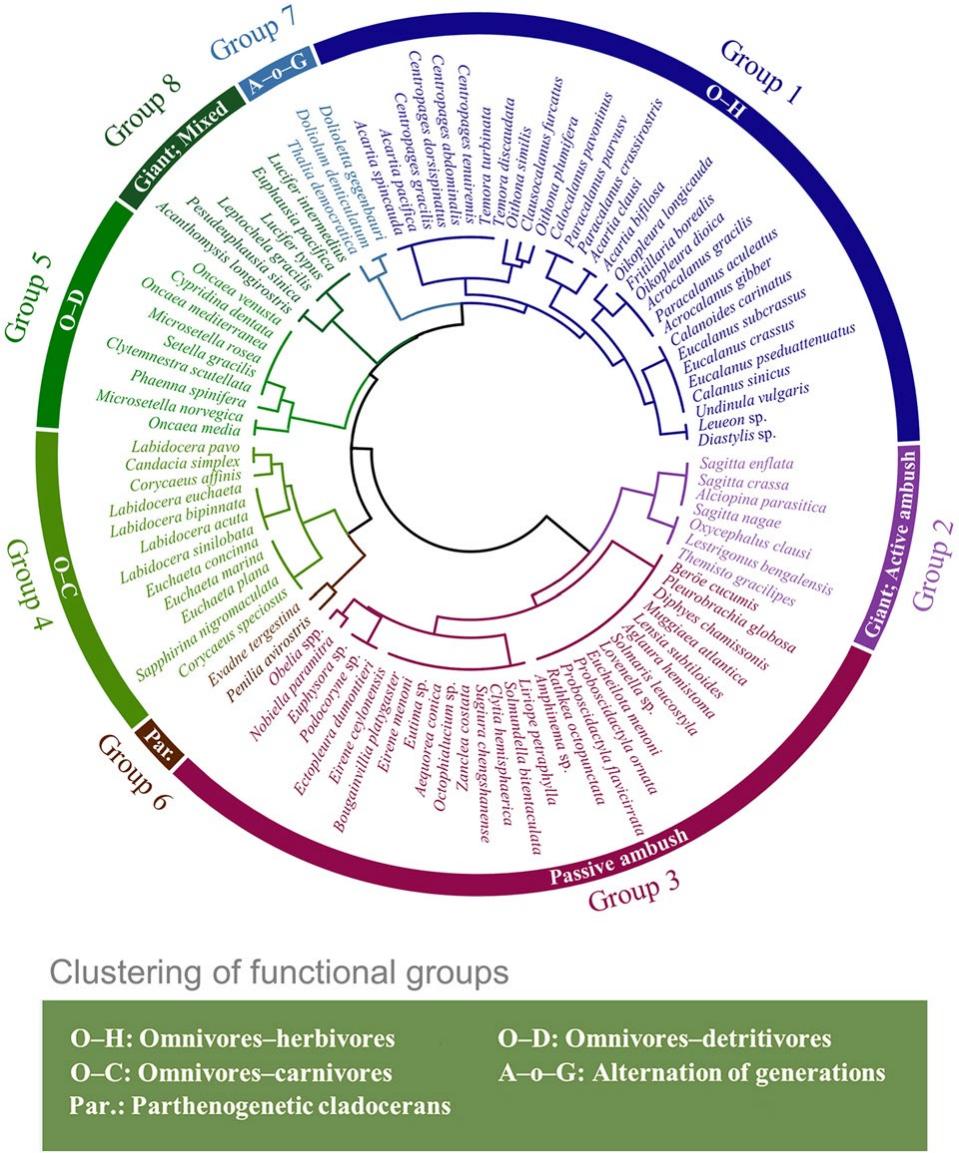
Dendrogram of zooplankton functional groups identified from hierarchical clustering based on four functional traits. Group 1, omnivores–herbivores (O–H); Group 2, giant active ambush carnivores; Group 3, passive ambush carnivores; Group 4, omnivores–carnivores (O–C); Group 5, omnivores–detritivores (O–D); Group 6, Parthenogenetic cladocerans (Par.); Group 7, alternation of generations (A–o–G) tunicates; Group 8, giant mixed crustaceans

**Fig. 5:**
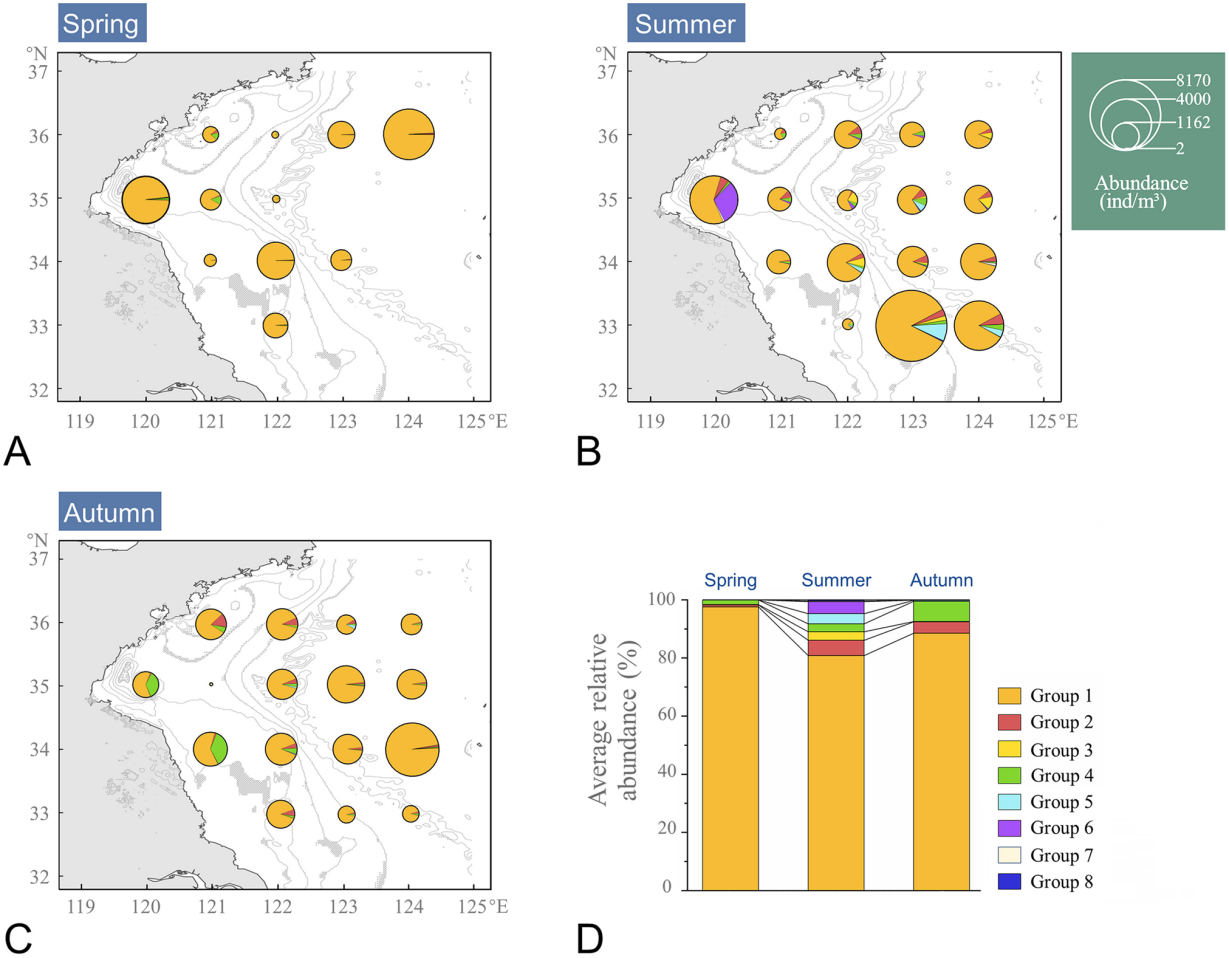
Biogeographic and seasonal variations of the zooplankton functional structure in the SYS. **A** spring; **B** summer; **C** autumn; **D** average relative abundance of three seasons

Agglomerative hierarchical clustering analysis was used to categorize identified species into eight functional groups according to their similarity in functional traits (Table 3, Fig. 4). Abundance contribution of individual functional groups showed strong seasonality and biogeographic variation (Fig. 5). Functional groups were most diverse in summer, followed by in autumn and spring (Fig. 5D), and was higher in nearshore than in offshore areas in autumn (Fig. 5C). Of the eight functional groups, omnivores–herbivores (Group 1) was the most abundant functional group accounting for more than 60% of total abundance and 81.0–97.6% with respective to seasons (Fig. 5). Carnivorous zooplankton were represented by Groups 2 and 3 and consisted of chaetognaths and gelatinous zooplankton (Hydromedusae, Siphonophorae, Ctenophores), respectively. Group 4 was composed of active ambush omnivore–carnivore copepods, and *Corycaeus affinis* contributed more than 90% to Group 4 abundance in all seasons. Group 5 consisted of omnivores–detritivores ostracods and copepods, while Group 6 was composed of two parthenogenetic cladocerans. Group 7 and 8, which contributed less than 0.5% of the abundance over all seasons, are represented by tunicates and malacostracans, respectively. Although the contribution of the remaining groups was much less than Group 1, seasonality was still significant. The proportions of Groups 2, 3, 5 and 6 all peaked in summer (2.9–6.9%), while that of Group 4 peaked in autumn (Fig. 5D). Moreover, spatial differences were also observed and the patterns varied among seasons, particularly for Groups 2, 4 and 6 (Fig. 5A–C).

**Table 3:** Summarized functional traits of the identified functional groups of zooplankton

Group	Species number	Taxa	Body length	Feeding type	Trophic group	Reproductive mode
1	30	Copepods, Cumaceans, Tunicates	Small, Medium, Large, Giant	Active ambush, Current, Mixed	Omnivore-herbivore	Free spawner, Egg-brooding
2	7	Polychaetes, Amphipods, Chaetognaths	Giant	Active ambush	Carnivore	Free spawner, Egg-brooding
3	29	Hydromedusae, Siphonophorae, Ctenophores	Small, Medium, Large, Giant	Passive ambush	Carnivore	Free spawner
4	12	Copepods	Small, Medium, Large	Active ambush	Omnivore-carnivore	Free spawner, Egg-brooding
5	9	Ostracods, Copepods	Small, Medium	Current	Omnivore-detritivore	Free spawner, Egg-brooding
6	2	Cladocerans	Medium	Active ambush, Current	Omnivore-herbivore, Omnivore-carnivore	Parthenogernesis
7	3	Tunicates	Medium, Large	Current	Omnivore-herbivore	Alternation of generations
8	6	Mysids, Euphausiids, Decapods	Giant	Mixed	Omnivore, Omnivore-carnivore	Free spawner, Egg-brooding

### Functional trait–/group–environment relationships

**Fig. 6:**
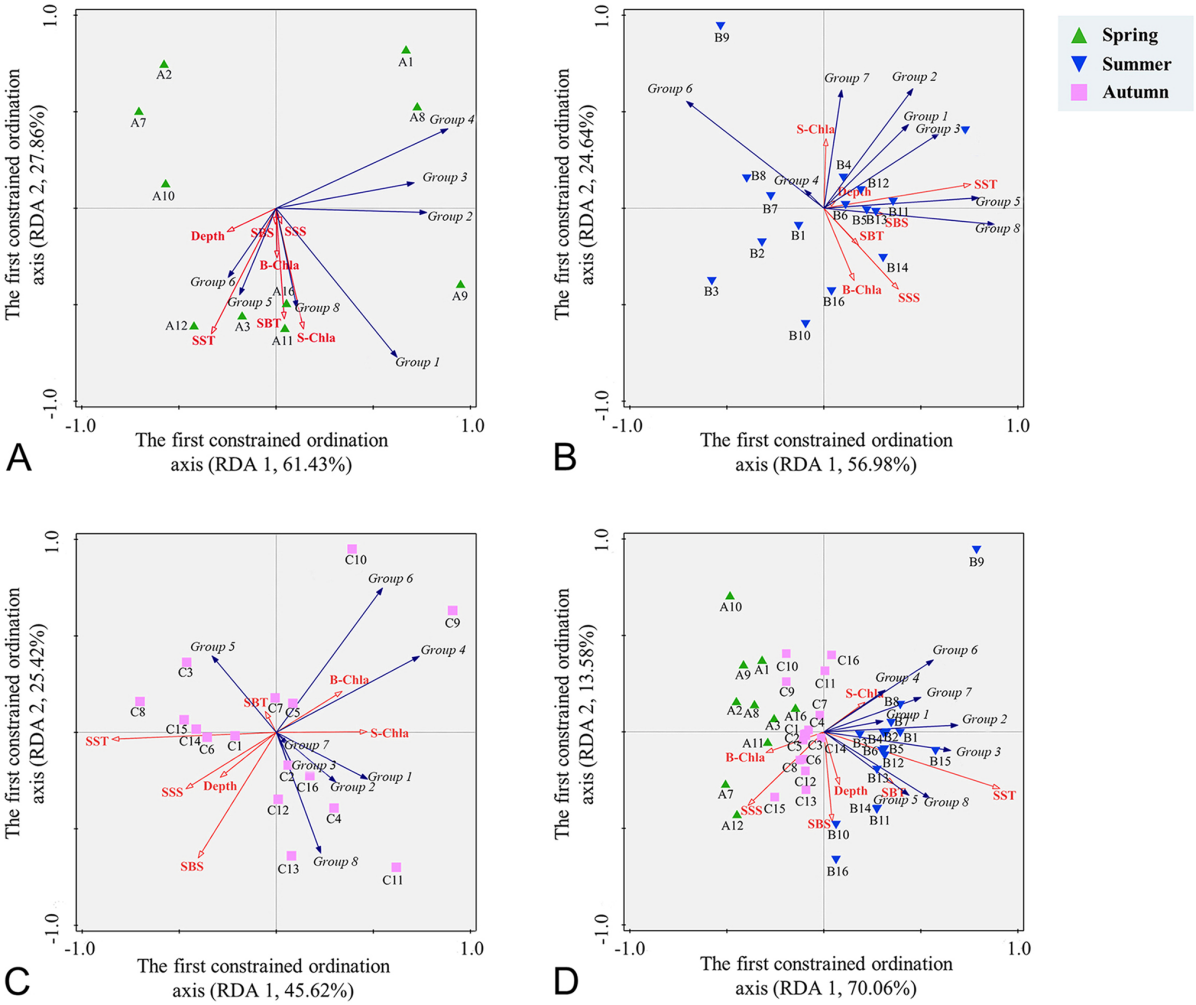
Redundancy analysis (RDA) bi-plot depicting the relationships between functional groups and main environment variables. **A** spring; **B** summer; **C** autumn; **D** three seasons. Green triangle: spring stations; orange triangle: summer stations; purple square: autumn stations

Redundancy analysis (RDA) showed that seasonal variations in functional traits and groups were both related to environmental factors. For functional traits, reproductive mode and feeding type were mainly influenced by temperature and phytoplankton biomass. Specifically, the abundance of active ambush and current feeders was positively correlated with S-Chla (Supplementary Fig. S3). The abundance of free spawners was positively correlated with SST and S-Chla, and that of egg brooders showed a positive correlation with SST and SBT (Supplementary Fig. S3). For functional groups, environmental factors explained the difference in zooplankton functional structure with a degree of 42.7% (Fig. 6D). Group 1 was positively correlated with SST and S-Chla (Fig. 6A and D). Groups 2 and 3 had a positive correlation with SST (Fig. 6D), and Group 2 showed a negative correlation with SSS (Fig. 6C and D). Group 4 showed a positive correlation with SST and S-Chla (Fig. 6D), and its horizontal distribution was positively correlated with Chl *a* in autumn (Fig. 6C). Group 5 was positively correlated with SST and SBT in all three seasons (Fig. 6D), and its horizontal distribution showed a positive correlation with SST in summer (Fig. 6B). Group 6 was positively correlated with SST and S-Chla (Fig. 6D), and its horizontal distribution showed a positive correlation with S-Chla in summer (Fig. 6C).

## Discussion

Functional traits are phenotypic characteristics of organisms that are relevant to ecosystem function (Violle et al. 2007). These features can be used to describe ecosystem dynamics and how they function with respect to environmental changes. In this study, we used a trait-based approach to quantify the seasonal variations of mesozooplankton functional structures in the SYS and found that the functional dynamics from traits to groups were closely related to environmental drivers, which will be discussed as below.

As the master trait, body length determines growth rates, swimming speed, fecundity, and the metabolism of zooplankton (Barton et al. 2013). In concert with previous studies in SYS (Li et al. 2022; Zhang et al. 2021), we also found that small zooplankton (< 1 mm), which are primarily driven by the relationship between the body size and temperature, were dominant in 2018 (Brun et al. 2016; Campbell et al. 2021; Evans et al. 2020; McGinty et al. 2018). Despite this, the relative contribution of each size class varied among seasons (Fig. 3A); this could be partially explained by changes in the size structure of the phytoplankton, one of the main prey types for zooplankton. In general, zooplankton size is positively correlated with prey size to ensure high feeding efficiency (Brun et al. 2016; Hansen et al. 1994). Unfortunately, there was no size-fractioned phytoplankton information (e.g., abundance, biomass) available for this study, but it has been well documented in other studies in SYS (e.g., Deng et al. 2008; Fu et al. 2010; Huang et al. 2006) that size structure is mainly driven by nutrient conditions. For example, it has been found that seasonal changes in nutrient conditions impact the shapes and size structure of phytoplankton and zooplankton in YSCWM (Fu et al. 2010; Huang et al. 2006; Huo et al. 2012; Shi et al. 2015; Wang et al. 2003). YSCWM is formed in spring, peaks in summer and gradually decays in autumn (Su and Weng 1994). In the spring, the solar radiation increases and rapidly warms the upper layer. Therefore, a strong seasonal thermocline forms quickly and reaches its peak in the summer at a depth of 10–20 m, which prevents vertical mixing (Yang et al. 2019). Therefore, oligotrophic surface water forms as a result of nutrient consumption in spring and reduced renewal of nutrient in the stratified water in summer. Oligotrophic waters are unfavorable for large-sized phytoplankton (> 2 μm) (Marañón et al. 2001; Fu et al. 2010), which consequently depresses the growth of the corresponding predators, the large-sized zooplankton in summer.

In terms of reproductive mode, free spawners were dominant in the SYS in all three seasons but peaked in autumn (Fig. 3D), displaying a positive correlation with S-Chla (Supplementary Fig. S3). Previous study on crustacean zooplankton in the SYS also revealed the dominance of free spawners, the dynamics of which were driven by hydrological seasonality (Li et al. 2022). It has been reported that free spawners dominate in coastal regions, for example, the coastal region of the Southwestern Atlantic (Da Conceição et al. 2021) exhibit high fecundity and egg-laying in environments with high Chl *a* (Bunker and Hirst 2004). Food availability and quality in essence determine the fecundity of the free spawners. Thus, the relatively high phytoplankton biomass in SYS (Deng et al. 2008) may favor the reproduction of free spawners. Moreover, the abundance of free spawners was positively correlated with SST (Supplementary Fig. S3). Bunker and Hirst (2004) pointed out that the fecundity of free-spawning zooplankton is positively correlated with temperature and the highest fecundity is detected at approximately 15 °C for most free spawners. In this study, the average sea water temperature in autumn was approximately 15 °C (Fig. 2C), which potentially resulted in the maximum abundance of free spawners over seasons.

As one of the main zooplankton traits, feeding strategies are critical for ecosystem functions, such as the transfer of energy and biomass to higher trophic levels (Prowe et al. 2019). The efficiencies of the various feeding modes are traded off against metabolic costs, predation risks, and mating chances (Kiørboe 2011). In this study, we observed significant seasonal changes in the composition of feeding types (Fig. 3C; Supplementary Table S1). Ambush feeding and current feeding zooplankton dominated in spring and autumn, respectively, which could be attributed to the seasonality of turbulence and food availability. Turbulence may increase the encounter probability between planktonic predators and prey, especially for ambush feeding zooplankton (Kiørboe and Saiz 1995). Compared with that in other seasons in the SYS, the vertical turbulent mixing is particularly strong in spring (Chen et al. 1980), and the high abundance of phytoplankton at that time also increases the chance of meeting their prey for ambush feeding zooplankton. In this study, the abundance of active ambush and current feeders showed positive correlations with S-Chla concentrations (Supplementary Fig. S3), pointing toward fluctuations in food availability being another factor driving the shift of feeding types over seasons. When Chl *a* concentration is low, active feeding zooplankton (e.g., current feeding) outcompete their passive counterparts (Prowe et al. 2019) by active cruising or generating a feeding current. By contrast, ambush predators can only passively encounter and intercept prey, which are favored under the high prey density and/or active prey-dominant conditions (Benedetti et al. 2016; Kiørboe 2011). Thus, the predation efficiency of active feeding species is higher than that of ambush predation in summer and autumn when low Chl *a* concentrations occur (Kiørboe 2011; Prowe et al. 2019); this is consistent with the results presented here. The number of offshore sampling stations with low Chl *a* concentration in spring was less than that in summer and autumn, which potentially contributed to the lower proportion of current feeding zooplankton being observed in spring. Moreover, the feeding types of zooplankton are also related to reproductive mode, with most of the free spawners being current feeders (Kiørboe et al. 2015), and most of the egg brooding zooplankton being ambush feeders (Benedetti et al. 2016). Our results are in accordance with previous findings as free spawners and egg brooders dominated in autumn and spring, respectively (Fig. 3D).

We further gathered taxa into functional groups and analyzed the seasonality of the functional structure of mesozooplankton with respect to environmental changes (Figs. 4 and 5). Functional group analyses simultaneously consider multiple traits and provide a comprehensive insight into ecosystem dynamics with response to environmental interference (Krztoń and Kosiba 2020). Of the eight functional groups, omnivores–herbivores (Group 1) were the most abundant (Fig. 5), consistent with previous finding in the Northeast Subarctic Pacific Ocean with omnivores–herbivores being the largest trophic group (Pomerleau et al. 2015). The abundance of Group 1 was positively correlated with S-Chla concentration and peaked in spring (Figs. 5D and 6D), indicating a close association with phytoplankton dynamics. Herbivorous zooplankton are commonly dominant in coastal waters and marginal seas where Chl *a* concentration is high (Mackas and Coyle 2005). By ingesting phytoplankton, herbivorous zooplankton transfer the energy fixed through photosynthesis to higher trophic levels (Søreide et al. 2006). Thus, the seasonal cycle of phytoplankton productivity and biomass greatly impacts the community dynamics of the herbivorous zooplankton (Behrenfeld and Boss 2014). The general processes are considered as follows (Behrenfeld and Boss 2014; Hu et al. 2004): in winter, the phytoplankton growth is limited by low light availability, but the nutrients are replete due to strong vertical mixing, setting the stage for the spring phytoplankton bloom. In spring, water-column stratification is restored by increased solar radiation and reduced wind, retaining phytoplankton in the sunlit surface waters. Consequently, spring phytoplankton blooms primarily provide food for herbivorous zooplankton (e.g., Group 1 in our study). With the development of YSCWM and the formation of a strong seasonal thermocline in the summer, a downturn in the phytoplankton bloom, and herbivorous zooplankton feeding occurs. Thus, a decreased abundance of Group 1 in the YSCWM areas in summer is observed (Fig. 5B).

Carnivore zooplankton in the SYS, represented by functional Groups 2 and 3, were strongly affected by temperature, salinity, and food availability (Figs. 5D and 6D). Group 2, mainly included carnivore chaetognaths, the distribution of which was strongly affected by temperature. This taxon tends to reside in the high-SST regions (Buchanan and Beckley 2015). In concert with our results of the functional group analysis, Dai (2006) also found that the abundance of chaetognaths in the SYS was higher in summer and autumn and dropped to its the lowest in spring, especially for *Sagitta enflata* (< 1 ind/m^3^), a warm-temperate species. However, due to the limited number of sampling stations in spring, the relative abundance of Group 2 may be underestimated. The horizontal distribution of Group 2 was affected by salinity in autumn (Fig. 6C). High abundance of chaetognaths is usually observed in productive waters with low salinity, such as estuaries (Noblezada and Campos 2012). Low salinity and sufficient food resources create a favorable habitat for the propagation of nearshore chaetognaths (Gilmartin et al. 2020). The YSCC is a low-salinity coastal current, which plays an important role in transporting nutrients southeastward (Wei et al. 2011). Meanwhile, the CDW, characterized by low salinity and high nutrient, enters the SYS from the Changjiang River estuary on a northeasterly trajectory (Wei et al. 2011; Wu et al. 2014). Thus, in the combined results of YSCC and CDW, the proportion of Group 2 was higher in nearshore than offshore areas in autumn, especially at H1 and H2 stations (Fig. 5C). Group 3, which was composed of carnivore medusa, positively correlated with SST (Fig. 6D). It has been considered that rising sea temperature could induce an increase in abundance of gelatinous plankton, such as cnidarians and ctenophores (Purcell et al. 2007). Temperature-induced physiological responses including growth, reproduction and metabolism, have been found in medusa, and increased temperature promotes the growth of medusa (Rosa et al. 2013). The rapid growth in the jellyfish population has been also detected during the summer in the SYS, when the average sea temperature exceeds 15 °C (Ma et al. 2000). Furthermore, food availability was another important factor regulating the population dynamics of jellyfish (Ma et al. 2000; Rosa et al. 2013).

Group 4 was composed of active ambush omnivore–carnivore copepods, with *C. affinis* contributing more than 90% to Group 4 abundance in all seasons. Group 4 was negatively correlated with SSS and SBS (Fig. 6). A study on the effects of sudden change in salinity on survival revealed that *C. affinis* cannot adapt to a salinity surge of over 4.8, likely because the high salinity adversely impacts osmoregulation (Jiang et al. 2009). The YSCC and CDW with low salinity may improve the survival rate of *C. affinis,* contributing to the higher proportion of Group 4 in the nearshore stations in autumn.

Detritivores are an essential component of marine biogeochemical cycles as they feed on detritus, such as carcasses and fecal pellets (Yamaguchi et al. 2002). Group 5 consisted of omnivores–detritivores. The abundance of Group 5 varied between seasons by over 35-fold, peaking in summer and displaying a positive correlation with SST (Figs. 5D and 6D). The population of planktonic detritivores relies on the abundance and activities of other plankton in the ambient waters, which provide the source of the detritus (Auel 1999; Pomerleau et al. 2015). Thus, environmental drivers mostly regulate plankton abundance and could indirectly affect the population dynamics of detritivores. For example, Zhang et al. (2010) suggested that zooplankton abundance is affected by sea temperature and that high abundance of zooplankton is associated with high water temperature. Thus, we postulate that the increased abundance of Group 5 in summer could be a result of the proliferation of zooplankton due to increasing SST.

Group 6 was composed of two parthenogenetic cladocerans, *Penilia avirostris* and *Evadne tergestina*, the abundance of which was related to temperature, Chl *a* concentration and position relative to the coastal current. Previous studies have reported that as temperature rose, the reproduction rate and population size of cladocerans increased rapidly and reached the peak in summer (Zheng et al. 1982); this is consistent with our findings (Supplementary Fig. S2D). As an omnivore–herbivore, *P. avirostris* contributed 94.4% to the abundance of Group 6. The coupling between *P. avirostris* and phytoplankton has previously been described in China’s nearshore waters (Zheng et al. 1982). In addition, the abundance of Group 6 in summer was higher in nearshore than offshore areas. *P. avirostris* is an indicator species of the location of the coastal current and its distribution is strongly controlled by the coastal current, which is mostly located in the nearshore, especially in low-salinity waters (Zheng et al. 1982). The waters of YSCC, with low salinity and high nutrients, is conducive to the growth of *P. avirostris* and this is reflected in the horizontal distribution of Group 6.

In this study, we observed spatial–temporal variations in both zooplankton taxonomy and functional structure, but patterns generated by the two approaches varied. At most stations, functional richness was closely associated with taxonomic richness (Supplementary Fig. S2; Fig. 5), indicating that diverse ecological strategies were adopted by different taxa. However, several stations (e.g., H6, H7 and H9) in summer, with higher taxonomic richness, presented equivalent functional richness to other stations or seasons. This suggests that environmental conditions in summer favored the high number of taxa but did not select specific ecological strategies, resulting in functional redundancy (Mouillot et al. 2013). High species diversity could be due to decreased competition among taxa, and higher functional redundancy is considered an indicator of resistance to environmental changes (Redmond et al. 2018). Moreover, we also found that the dynamics of the dominant species were more complex than that of the functional groups. For example, the average abundance of different components in Group 1 showed distinct seasonal patterns, with *C*. *dorsispinatus*, *A*. *pacifica*, and *O*. *plumifera* peaking in summer, while *C*. *abdominalis* peaked in spring (Table 2). Despite this taxonomic variation, the dominancy of Group 1 persisted over seasons (Fig. 5), pointing toward functional stability in SYS.

Studying biodiversity is critical to understand the interplay between zooplankton community and the functioning of marine ecosystem. In particular, the functional diversity and redundancy within a community can be exploited to simulate and predict the impacts of environmental change (Mouillot et al. 2013; Norberg 2004). This can be achieved using ecological modeling, which relies strongly on the specific traits governing biological processes (e.g., food web interaction, types of life history and resource acquisition), rather than taxonomy (Litchman and Klausmeier 2008). By identifying the trade-offs between functional traits/groups and quantifying their relationships with physiological and biochemical changes, ecosystem models can simplify the contribution of species to understand the complex ecological processes and how the real ecosystem might respond. However, zooplankton information in current models is usually parameterized based on sporadic data from laboratory or limited field studies (Barton et al. 2013) with only size class considered (Flynn et al. 2015; García-Comas et al. 2014; Le Quéré et al. 2005). Although size is the master trait of an organism, relying on it alone will definitely oversimplify the community functions and ecosystem responses (Flynn et al. 2015). As identified in several modeling studies, in the context of global biogeography and biogeochemical cycles under climate change, simultaneously considering multiple traits in modeling is crucial to clarifying the contribution of species and identifying the environmental determinants (e.g.,Benedetti et al. 2022; Prowe et al. 2012). Therefore, investigating the link between zooplankton diversity and ecosystem function using a trait-based approach will improve the representation of zooplankton in global marine ecosystem models and enhance the model predictability and interpretation.

We acknowledge several technical limitations and difficulties associated with sampling in this study. Planktonic larvae and ontogeny were not considered in the trait analysis due to the difficulty in identifying them to species level and limited information of traits. To the best of our knowledge, existing datasets of zooplankton trait mainly consider only mature stages (Barnett et al. 2007; Benedetti et al. 2016). In our study, the relative abundance of planktonic larvae was low (averagely < 10%), thus the exclusion of larvae had little impact on the validity of the functional analysis. Zooplankton were sampled with a 200 μm mesh size WP2 net. Therefore, the abundance of small copepods, an important part of mesozooplankton, may be underestimated (Riccardi 2010). In the field, when strict consistency of sampling time (day or night) at different stations could not be guaranteed, the abundance of net-collected plankton might have been affected by the diel vertical migration of zooplankton (Heywood 1996). Furthermore, there were less sampling stations in spring and no winter sampling available in our study. Thus, year-round sampling is required in future to portrait season by seasonal patterns in the biogeography of zooplankton functional structure.

## Materials and methods

### Study area and sampling

Sampling was carried out by performing three surveys of the SYS (33–36 °N, 120–124 °E) in 2018 onboard R/V KEXUESANHAO for 11, 16, and 16 sampling sites on April 18 to 22 (spring), August 29 to September 6 (summer), and November 19 to 27 (autumn), respectively (Fig. 1).

Zooplankton samples were collected by vertical tows using a WP2 plankton net (mouth area: 0.25 m^2^, mesh size: 200 μm) from about 3 m above the bottom to the surface. The collected samples were stored in formalin–seawater solution with a final concentration of 5%. Preserved samples were examined by stereoscopic microscope (SZM-LED2, OPTIKA) to identify the zooplankton community morphologically. In situ temperature and salinity were obtained using a shipboard rosette-mounted Conductivity-Temperature-Depth casts (CTD, Sea Bird 911) with the probe of temperature and conductivity, respectively. About 500 ml seawater was collected from surface and bottom layers, respectively, for chlorophyll *a* (Chl *a*) measurement. Seawater was filtered through GF/F membrane (Whatman) and stored in liquid nitrogen until analysis. Chl *a* concentration was determined in the laboratory using a UV fluorescence spectrophotometer (F-4500, Hitachi, Japan) after extraction with 90% acetone for 24 h under 4 °C (Shi et al. 2018).

### Functional traits and groups identification

Four major functional traits were analyzed for each taxon including: (i) *average adult body length* of small (< 1 mm), medium (1–2 mm), large (2–5 mm), and giant (> 5 mm) (Sun et al. 2010); (ii) *feeding types* of active ambush feeding, passive ambush feeding, current feeding, and mixed feeding for species that could switch between two types (Kiørboe 2011); (iii) *trophic groups* of carnivores, omnivores, omnivores–carnivores, omnivores–herbivores and omnivores–detritivores (Benedetti et al. 2016). ‘Omnivores–carnivores’ refers to mainly carnivorous species that sometimes feed on organic detritus or other small organisms. ‘Omnivores–herbivores’ refers to primarily herbivorous species that occasionally eat other small organisms or organic detritus. ‘Omnivores–detritivores’ refers to species that feed mostly on organic detritus and occasionally eat phytoplankton; (iv) *reproduction modes* of free spawner, egg brooding, parthenogenesis, and alternation of generation (Li et al. 2022).

Information on the traits of each species was obtained from the literature (Barnett et al. 2007; Benedetti et al. 2016), public datasets including Encyclopedia of Life (http://www.eol.org) and Marine Planktonic Copepods (http://copepodes.obs-banyuls.fr/en). While limited by the information available, the trait assignment to each zooplankton species was based on mature stages, so trait modality of certain species in ontogeny were not considered and was assumed to be unchanged throughout the year. The species were classified according to the extent to which they displayed the categories of each biological trait using “binary” coding. Traits exhibited by species were assigned a value of “1”, and traits not exhibited by species were assigned a value of “0” (Supplementary Table S2). Finally, a species × trait matrix was generated for downstream analysis (Zhong et al. 2020).

Functional groups were clustered followed Krztoń and Kosiba (2020) using “Factoextra” package in R (version 4.1.0). The data were arranged according to the species × trait data matrix and imported into R (version 4.1.0), on which the dissimilarity matrix was calculated with Gower distance. Ward’s agglomerative hierarchical clustering method (Ward 1963) was used to classify species according to their similarity/difference in functional traits. Briefly, with the dissimilarity matrix calculated above, two species are grouped together when it minimizes a given agglomeration criterion. Then the dissimilarity between this cluster and the rest species is calculated to generate clusters that have minimum within-cluster variance. This process continues until all the species have been clustered. Finally, the Elbow method was applied to determine the optimal number of functional groups (clusters) (Kassambara and Mundt 2017).

### Statistical analysis

Zooplankton abundance was standardized to individuals per m^3^ (ind/m^3^). The standard deviation was used to reflect the dispersion of zooplankton abundance. Dominant species were identified according to dominance indicator, which was calculated as *Y* = (*n*_*i*_/*N*) × *f*_*i*_, where *Y* ≥ 0.02 is the dominant species. Analysis of variance (ANOVA) was carried out to test for differences in taxa and dominant species between three seasons. ANOVA was conducted using SPSS 25 software. The relationship between functional groups and environmental variables was examined by RDA in CANOCO 5.0. Prior to this test, the abundance of all functional groups was log (*x* + 1)-transformed. The Wilcoxon rank-sum test was used to examine seasonal differences in each functional trait. The voyage station map and horizontal distribution of environmental parameters were created by Ocean Data View 5.2 software.

## Supplementary Information

Below is the link to the electronic supplementary material.

Supplementary file1 (DOC 2721 KB)

## Data Availability

All data generated or analyzed during this study are included in the manuscript and supporting files.
